# A hidden ambiguity of the term “feedback” in its use as an explanatory mechanism for psychophysical visual phenomena

**DOI:** 10.3389/fpsyg.2014.00780

**Published:** 2014-07-22

**Authors:** Talis Bachmann

**Affiliations:** Institute of Public Law, University of TartuTartu, Estonia

**Keywords:** awareness, reentrant processing, feedforward processing, non-specific thalamocortical modulation, masking

Over the last decades many researchers have used concepts like “feedback,” “reentrance,” “backpropagation,” “top–down (modulation),” or “reverse hierarchy” to specify the mechanisms that underlie various visual phenomena (e.g., Di Lollo et al., 2000; Lamme and Roelfsema, 2000; Pascual-Leone and Walsh, 2001; Supèr et al., 2001; Ro et al., 2003; Ahissar and Hochstein, 2004; Bar et al., 2006; Fahrenfort et al., 2007; Koivisto, 2012). An incomplete list of these phenomena includes visual (object substitution) masking, shape discrimination, illusory contours, illusory motion, priming effects, etc. Empirical evidence or theoretical argumentation in favor of the suggested mechanismic explanations mainly consists in finding or postulating an association between a temporally delayed, secondary activition of lower level neural units with correct reports of target stimuli, even though the higher level neural units in the processing hierarchy were already activated earlier. On that basis, feedforward processing has been argued to be insufficient for target perception. However, in most of the studies the relative *temporal order* of activity at different levels alone is taken as proof of reentrant modulation without precisely measuring the *neural sources* of this top–down effect. In principle, it is equally possible that the source of the higher level activity from which the top–down signals are sent back to earlier feature-encoding neural units (i) is specifically linked to those features by virtue of constituting the higher level nodes associated with specific attributes of the target stimulus (thus mediating feature-binding for object integration) or (ii) is not specifically linked in this manner. In the latter case, the *source* of top–down modulation may be the result of the arousal or alerting boost triggered by the target stimulus via feedforward collateral activation of subcortical reticulo-thalamic units, which in turn is followed by the cortical spread of the thalamocortical activation, including the downpropagation of the non-specific wave of modulation to the early cortical areas. The non-specific system functions include arousal, attentional modulation, intercortical synchronization of neural activity, bringing the preconsciously processed specific content to awareness, “event-holding” the content in working memory, and alerting subjects to newly appearing objects and changes (Magoun, 1958; Purpura, 1970; Purpura and Schiff, 1997; Jones, 2001; Llinás and Ribary, 2001; Van der Werf et al., 2002; Ribary, 2005; Schiff et al., 2013; Saalmann, 2014). This non-specific system (NSP) targets layer-1 apical dendrites of the layer-5 and -6 pyramidal neurons. But since NSP-modulation is directed at the cortical neurons with specific representational functions, its function may go unacknowledged because the cortical units, when activated by NSP-modulation, can produce content-specific subjective effects misleading us to believe that the entire process has been specific throughout.

The focus of the present paper will be on the experimental-behavioral and neurobiological evidence in comparing the two processing modes, (i) and (ii), with arguments from computational modeling left for some other occasion.

It is known that reticulo-thalamic, intralaminar and other matrix cells of the NSP project more heavily to lateral and frontal cortical areas and less so to the primary visual areas. (Even when rare examples of direct intralaminar-thalamic input to V1 were documented, these afferents were found to be much sparser than the more frontal ones—Miller and Benevento, 1979.) Moreover, this more rostrally directed thalamo-cortical flow can cause cortical responses as fast as or even faster than the afferent volleys through the specific geniculo-cortical pathways ignite primary visual cortical responses strongly enough (Kennedy and Baleydier, 1977; Kaufman and Rosenquist, 1985; Herkenham, 1986; Cruikshank et al., 2012; Liang et al., 2013; Saalmann, 2014). Thus, the primary cortical areas receive NSP-modulation not directly, but via the higher level cortical neurons that project onto apical parts of the layer-5 pyramidal neurons in the lower cortical areas. Consequently, as illustrated in Figure 1, we have two principal modes through which lower level neural units L responsible for encoding sensory features of perceptual objects receive top–down input from higher levels H: (i) from the specific nodes in H that were previously activated by L in a cortical feedforward manner and that now send reentrant signals back to L (here the feedforward-reentrant loop pertains to the specific sensory-perceptual attributes constituting a perceptual object LH); and (ii) from the generic nodes G that were activated by the boost of the NSP directed at the more frontal and mid-level cortical neurons that now send their downpropagating wave to the lower level visual areas, including L.

**Figure 1:**
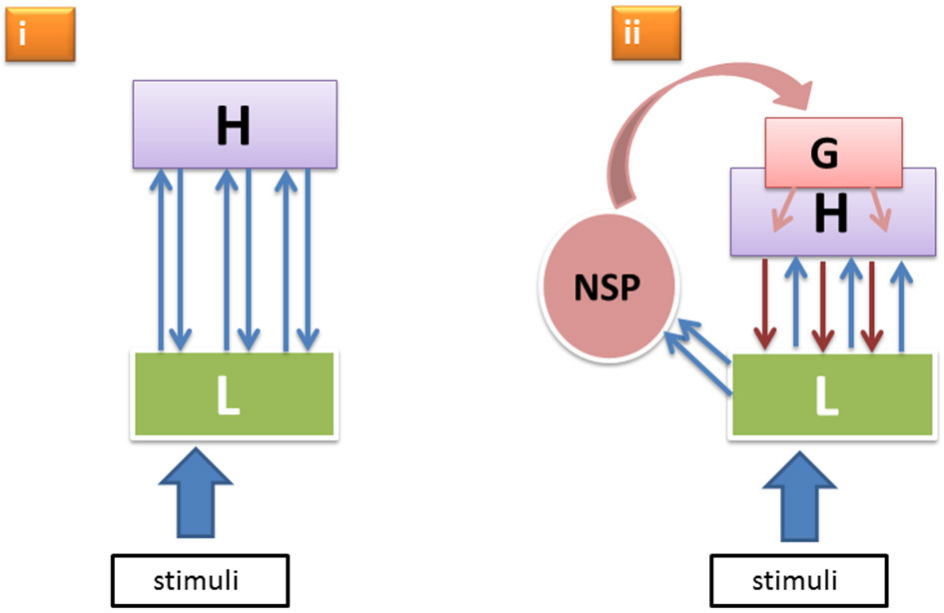
**Two alternative modes (i, ii) of top–down effects within the hierarchical perceptual processing system featuring specific low level neural units L, higher level specific nodes H, and a generic non-specific activation G boosted by the non-specific thalamocortical afference from NSP**.

When analyzing the experimental data from most of the studies that propose *specific* top–down linkages (i), there is no direct evidence that would invalidate the alternative, *non-specific* theory of downpropagation (ii). The specificity of visual experiences is due to the fact that the NSP-modulation arrives at specific early units L and may not be due to the specificity of the higher level from where this modulation arrives. Although the direct input from NSP to L may be weak, the top–down input from higher levels H/G driven by NSP may be strong enough to emphasize the specificity of the visual experience encoded in L. The pending task should be to try disentangle these two explanations experimentally. The experiments should ascertain whether the two modes of top–down modulation are incompatible or mutually complementary. In the latter case—how the two types of downpropagation are specifically combined and what relative roles each of them has? It is also possible that the standard views of reentrance (e.g., Di Lollo et al., 2000; Lamme and Roelfsema, 2000) may be valid in some empirical instances, difficult to ascertain in some other cases, and incompatible with the neurophysiological realities of processing in different experiments. Let me comment on some examples of typical experiments aimed at supporting the standard views of reentrance listed below and see whether version (i) should be exclusively preferred or whether versions (i) and (ii) both are compatible with the experimental results.

1. In typical object substitution masking (OSM) a target stimulus (e.g., a Landolt C) is presented together with four dots that surround the target. When after a very brief delay the target is switched off, the four dots either are also switched off or remain displayed for varing duration acting as a post-mask (the simultaneous onset, asynchronous offset condition.) The delayed-offset condition leads to strong masking but in the simultaneous-offset condition masking is weak. The classic theory of OSM (Di Lollo et al., 2000, but see Põder, 2013) explains this by a reentrant model (a variety of model i) according to which target-activated units at level H activated by the target send reentrant signals back to level L in order to test whether levels H and L are consistent in representing the target. If mismatch is registered (e.g., when target signals do not arrive anymore and mask signals arrive instead), the iterative feedforward-reentrant cycles are interrupted and new iterative “hypothesis testing” begins for the new object—the mask. Because cycles of reentrance are necessary for registration of the stimulus in awareness, the target is not consciously perceived when reentrant testing is prematurely interrupted by the stronger top–down mask signal. However, when mask's offset is synchronous with that of the target, the target-plus-mask is a composite object that provides both level L and H contents; hence, the target can be extracted from the composite representation that is maintained through the feedword-reentrant cycles. Let us see how the model (ii) works for OSM. Presentation of target evokes specific signaling along L-H vertical axis and also a collaterally ignited boost of NSP modulation. (NSP is necessary for awareness of the specific contents represented by L and H.) When asynchroneous-offset mask remains in view and target signals do not arrive anymore, the top–down activation G that was initiated fast at higher levels, but takes time to become active at lower levels “finds” mask related activity in L, but the target related activity has decayed already realtive to the mask activity, because the target was switched off earlier. Although the level G activity is non-specific, when its downpropagating generic influence reaches L it helps emphasize mask features because level L units themselves *are* specific. The mask-object representation becomes consciously perceived instead of target. Thus, models (i) and (ii) both are usable. At this point one may ask why not follow Ockham's rule and take the simpler one (i), i.e., the one with fewer hypotheses? However, the G units are important because neurobiological evidence has overwhelmingly shown that NSP is necessary for awareness of the specific contents represented by L and H.
2. In Lamme et al. (2000) monkeys were trained to discriminate visual targets. V1 responses began to differentiate the “seen” from the “unseen” trials after 125 ms. In subsequent studies occipital ERPs in humans differentiated visibility of masked targets after 109–141 ms or peaked at about 160 ms (Fahrenfort et al., 2007, 2008). Again, a variety of model (i) was used for explaining the results because specifically the temporally late target related activity at level L (which followed earlier time epochs sufficient for level H to have become active in target processing) were associated with correct discrimination. And again, model (ii) can explain these empirical results: the late part of neural activity at L which is enhanced in trials where target is successfully discriminated may be modulated by the top–down process G passed down through levels H (or even bypassing stimulus-specific level H units either via direct fibers or level H units different from the stimulus-related ones).
3. Temporal precedence of high-level MEG activity which discriminated correct and incorrect target processing over low level activity in the study by Bar et al. (2006) was also interpreted as a variety of model (i). However, if activity of G at frontal sites fluctuates (fluctuation of the thalamocortical NSP activity is a norm rather than an exception) and dictates whether the top–down modulation is stronger or weaker, these experimental results can be interpreted also according to model (ii).
4. Ro et al. (2003) utilized transcranial magnetic stimulation (TMS) in a metacontrast masking paradigm and showed that TMS of visual cortex, when timed to produce visual suppression of an annulus (a metacontrast mask), induced recovery of a target disc which was imperceptible when TMS was not used. Moreover, TMS suppression of an annulus was more pronounced when a disk preceded it than when an annulus was presented alone. The authors assume that when the later activity, supposedly reflecting the reentrant effects is suppressed then target perceptibility can be reinstated. They argue that a prior visual stimulus can influence subsequent perception at early stages of visual encoding via feedback projections, supporting model (i). Alternatively, model (ii) can also be applied. It is known that a preceding brief stimulus (e.g., target) speeds up perception of the following stimulus (e.g., mask) (Bachmann, 1989; Scharlau, 2007). When target disc was presented before mask it may have speeded up masking annulus processing by presetting NSP modulation for its signals. This in turn may have optimized the effective processing delay so as to coincide with the maximal TMS effect.
5. Up to now, both models appear to be equally applicable, but model (ii) provides an explanation of the results of an elegant experiment carried out by Wu et al. (2009) that model (i) cannot as readily provide. Capitalizing on the motion-induced blindness (MIB) phenomenon (Bonneh et al., 2001), where a static visual target-object continuously presented on a rotating background periodically disappears from awareness, they showed that a flashed stimulus that *caused* reappearance in awareness of the target was perceived *after* the reappearance of the target in consciousness. (The temporal value of reversal was about 100 ms, which is the value assumed to characterize the full cycle of reentrance based visual processing for awareness.) The temporal advantage of updating the conscious representation from the preexisting unconscious representation of the invisible static target was explained by a version of model (i), invoking reentry of neural signals after the first feed-forward sweep for a stimulus to be consciously perceived. Thus, MIB, by blocking reentry signals, prevents awareness. In Bachmann and Aru (2009) we pointed out some inconsistencies of this explanation and offered an explanation in terms of model (ii). When an object fades from awareness by MIB, its L and H level activity will be sustained because cortical specific signals are constantly present, but now it is dissociated from NSP-activity. When the flashed object is presented, the L/H process for representation of the flash occurs in parallel with a boost of the NSP-process igniting G. G leads to binding of the already present pre-conscious L/H-activity of the target with global consciousness-level representation. This process takes little time, because there is no need for build-up of the content-specific L/H representation of the target; consequently, its rapid reappearance in consciousness. The flashed object appears in consciousness not as fast, because its corresponding coherent L/H-representation must be built up, which takes time. The G that services *target* awareness has L/H content of the target ready on the “waiting list” but the G process has to wait as a “dummy process” until the L/H contents of the *flashed object* are ready to be modulated.

It appears that experiments have difficulty in distinguishing between the two models. This raises the question whether a computational/mathematical argument could be developed that allows to test different predictions about experimental data on the basis of the two models. Sadly, space does not allow me to dwell into this important perspective which must be dealt with in future research.

## Conclusion

In this opinion paper I argued for the view that in the majority of the standard experimental studies set to support the model of top–down processing featuring exclusively the specific system components also the combined non-specific/specific model seems equally valid.

### Conflict of interest statement

The author declares that the research was conducted in the absence of any commercial or financial relationships that could be construed as a potential conflict of interest.
